# Fungus Holds Clues to the Evolution of Sex Chromosomes

**DOI:** 10.1371/journal.pbio.0020435

**Published:** 2004-11-09

It's a basic biological principle that living things share certain fundamental traits. That's why understanding the mechanisms of cell division in single-celled yeast, say, can offer insight into cell division in humans. Now Joseph Heitman and colleagues report that the evolutionary events that spawned sex chromosomes in yeast resemble those that shaped sex chromosomes in animals.

Strictly speaking, yeast—the common name for single-celled fungi—don't have sex chromosomes; they have sex-determining regions within chromosomes, called mating type, or MAT, loci. In a comparative genomic analysis of the MAT locus in three species of the human pathogenic fungus Cryptococcus, Heitman and colleagues found that this fungal sex-determining region arose via a series of discrete events that echo those that shaped mammalian sex chromosomes.

A primary benefit of sexual reproduction is the genetic diversity gained from reshuffling genetic material during meiosis, which creates gametes. Yeast sex, such as it is, accomplishes the same thing. Of course, sexual identity for a fungus does not take the form of sperm or egg but of mating type **a**, for example, and mating type alpha. Still, yeast manage a measure of complexity and considerable elegance in the systems they deploy to sexually reproduce.

In ascomycetes, like baker's yeast, the MAT locus is small and includes just a few genes. The genes that determine a cell's **a** or alpha mating status are alleles (variants) of a single MAT locus. Cells with the MAT**a** allele are mating type **a**, while cells with the MATalpha allele exhibit mating type alpha. A cell can switch its mating type when genetic exchange, or recombination, between two mating loci occurs.[Fig pbio-0020435-g001]

**Figure pbio-0020435-g001:**
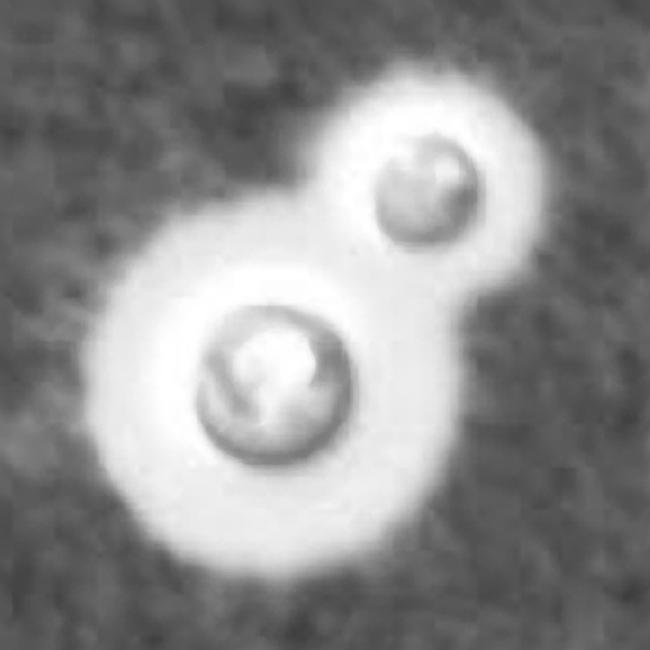
Human pathogenic fungus Cryptococcus

In basidiomycetes, like the corn smut Ustilago maydis—a maize pathogen that some consider a culinary delicacy—mating is more complex, and sexual identity is determined by two unlinked genomic regions with distinct classes of genes. Cells must be of different mating types at both loci to allow sexual reproduction. To their surprise, Heitman and colleagues discovered that the mating locus of Cryptococcus neoformans—a basidiomycete fungus that infects humans and is associated with transplant recipients, patients with AIDS, and other immune-compromised patients—exhibits several unique features, common to neither ascomycetes or their basidiomycete relatives.

Unlike most basidiomycetes, the C. neoformans locus occupies a single region and is unusually large, spanning more than 100 kilobases and containing over 20 genes, including those typically segregated in separate locations in other basidiomycetes. Like on the human Y chromosome, the sex-determining genes of C. neoformans are interspersed with non-sex-related genes. And unlike ascomycetes, which also have a single active MAT locus and two mating types, no mating type switching occurs as there are no silent mating type cassettes in the genome.

Heitman and colleagues sequenced the **a** and alpha alleles of C. neoformans' closest relative, C. gattii, and compared these variants to four already characterized variants derived from two C. neoformans subspecies. All six MAT alleles share characteristic features, including a fairly large size, a common gene set, and dramatic genomic migration during evolution (which is unusual compared to other genomic regions in the three strains). Each MAT allele has genes with different evolutionary histories, ranging from ancient to recent, that fall into distinct patterns based on shared nucleotide composition and mating type. The patterns correlate with how long the genes have occupied the MAT locus, suggesting how it evolved.

The authors hypothesize that this novel structure was formed by chromosomal rearrangements that linked two unrelated genomic regions into a single region. Recombination between these sex-determining regions was suppressed after other events blurred their boundaries. Specific genes in the once separated loci then attracted mobile elements in the genome to their sites, thus precipitating expansion of the locus. Because the Cryptococcus MAT locus resembles the evolution and structure proposed for the ancient Y chromosome, the authors argue that Cryptococcus can serve as a valuable model to study the molecular dynamics of sex chromosomes.

